# Modern Home Cooking Practices, the Role of New Media, and Implications for Culinary Medicine: A Qualitative Study Among Mothers With Low Income

**DOI:** 10.1177/15598276231197181

**Published:** 2023-08-24

**Authors:** Margaret Raber, Maria Vazquez, Syeda Khan, Sahiti Myneni, Debbe Thompson

**Affiliations:** 1 4002 University of Texas MD Anderson Cancer Center, Houston, TX, USA (MR, MV); 2 44249 USDA/ARS Children’s Nutrition Research Center, Baylor College of Medicine, Houston, TX, USA (MR, MV, SK, DT); 3 14743 University of Houston, Houston, TX, USA (SK); 4 UTHealth School of Bioinformatics, 182519 University of Texas, Houston, TX, USA (SM)

**Keywords:** qualitative research, new media, technology, low-income, culinary medicine, nutrition

## Abstract

Culinary medicine offers a practical, experiential approach to nutrition education, but in-person programs are resources intensive. Digital interventions may offer a scalable, acceptable approach to culinary medicine in populations that are at increased risk for poor diet, such as parents with low income. The purpose of this study was to examine modern home cooking behavior and the role of new media from the perspective of parents with low income and identify implications for culinary medicine research. Twenty parents from 6- to 11-year-old children that qualify for free/reduced school lunch programs completed a survey and interview examining online cooking information seeking behaviors, current cooking practices, and factors that influence healthy eating. Interview transcripts were analyzed using a semi-structured hybrid coding approach. Three major themes emerged from the data: (1) Current cooking habits and environment; (2) Factors that influence healthy cooking; and (3) The role of the internet in home cooking. This research may be used to inform the creation of digital culinary medicine intervention tools to promote healthy eating in this population.

“Acknowledging information quality as an issue is important when attempting to develop health promotion materials that rely on new media.”

## Introduction

Diet is a key modifiable risk factor for cardiometabolic disease and several cancers.^1–3^ Diet quality is low across the U.S. population,^4,5^ with mean Healthy Eating Index (HEI) scores particularly low among school-aged children, and those in lower-income households.^6,7^ The majority of children and adults in the US do not meet recommended intakes for fruit, vegetables, and whole grains^5–7^; and consumption of processed/red meat and ultra-processed foods is particularly high among families with low income.^7,8^ Dietary habits established in childhood track into adulthood and influence future risk for diet-attributable disease.^9–11^

Parents are key agents of influence on diet as they typically control how food is purchased and prepared.^12^ Families with low income have developed unique foodways (the multifaceted structures of food sourcing, preparing and eating behaviors that are influenced by familial arrangement, preferences, cultural norms, and food access) to cope with limited resources.^13^ For example, adults with low income prepare food at home more often and spend more time cooking than those with higher incomes.^14–17^ Overall, preparing and consuming foods at home has been associated with lower food costs^18^ and better diet quality among adults and children,^17,19–22^ while meals prepared outside the home are associated with poorer food choices.^23–25^ The degree to which diet quality is associated with cooking frequency, however, is significantly lower among Americans with low income compared to those with higher incomes.^16,26^

Improving parent cooking practices may increase diet quality among children living in low-income households by cultivating strategies to encourage healthy eating in the context of existing foodways, as opposed to promoting radical shifts in consumption that may not be attainable or sustainable.^27^ While culinary medicine programs are increasingly popular approaches to nutrition education, most rely on in person learning, which may be out of reach for lower resourced communities. Digital culinary medicine programs may offer a more scalable approach to practical nutrition education. The internet and smartphone applications have emerged as important sources for recipes among parents with low income^28,29^ and over one-third of respondents in a nationally representative survey reported learning to cook from online resources.^30^ Little is known about how these new media sources (any digital media including internet, social media, smartphone apps, etc.) influence home cooking behavior in families with low-income. Understanding of the role of new media in home cooking behavior will support the creation of scalable culinary medicine tools for this high-need population. The objective of this study was to qualitatively examine modern home cooking behavior and the role of new media from the perspective of parents from 6- to 11-year old children with low income and identify implications for culinary medicine research and practice.

## Materials and Methods

This study was reviewed and obtained ethics approval from the Baylor College of Medicine Institutional Review Board (H-50324). Participants completed an informed consent document before taking part in study procedures.

### Participants

Parents from 6- to 11-year old children from low-income households in the Houston, TX, Metropolitan Statistical Area^31^ were recruited for this study. Low-income was defined as qualifying for free or reduced school lunch programs (i.e., household income is ≤185% of the federal poverty line). Additional inclusion criteria were English-speaking, report cooking at least three times per week on average, and report having some familiarity with online recipes. Online recipe familiarity was assessed with the question “How often do you look at online recipes?”, and those that responded “never” were excluded. This was to ensure an information-rich sample on the topic of cooking and new media. Recruitment took place over eight weeks and was done through flyers in the community and via an email list-serve maintained by the USDA’s Children’s Nutrition Research Center. After completing an online screener, eligible participants reviewed and completed an informed consent document and were enrolled.

### Data Collection

Enrolled participants were sent a link to a brief online survey that included demographic questions as well as items covering online recipe/nutrition information seeking behavior, current cooking practices, attitudes about cooking and health, and factors that influence healthy cooking and eating. The survey responses were used to guide the subsequent interviews (e.g., “you stated on the survey you look at online recipes *every day*, what are some reasons you look at online recipes that often?”). Using open-ended questions, interviews went into further depth regarding perceptions of new media usage and its influence on home cooking behavior. Interviews lasted up to one hour and took place via videoconference (audio only). Interviews were recorded, professionally transcribed, and transcriptions were quality checked against the audio prior to analysis to ensure accuracy. Following the interviews, all participants were compensated $25 for their time.

### Analysis

Descriptive statistics were used to assess survey response. Interview transcripts were analyzed using Dedoose software. All coders listened to interview recordings prior to coding the transcripts which served both as a quality check to the transcription and allowed coders to better familiarize themselves with the material. A semi-structured hybrid coding approach was used to code the data. This process included 5 main steps: (1) A summary analysis of each interview was created to identify responses to the interview questions. This set the foundation for deductive primary codes; (2) One coder listed all responses identified in the interview summaries for each key concept extracted from the interview questions; responses that were mentioned in more than one transcript formed deductive subcodes. Once complete, a second coder reviewed the interview summaries and discussed further changes to the subcodes. Discrepancies were mediated by the first author. (3) Deductive primary and subcodes were crafted into a codebook with formal definitions, rules, and example quotes. (4) The codebook was then applied to the interview transcripts. Three study staff applied the codebook to all interview transcripts and added new inductive primary and subcodes as they emerged. By the sixth interview, no new inductive codes were added and the codebook was stabilized with both deductive and inductive primary and subcodes. (5) Two coders then independently re-coded all the interviews using the finalized codebook. No new codes were added during this process, suggesting codebook saturation at 6 interviews in this sample, which is in line with other phenomenological research.^32–34^ Discrepancies between coders were mediated through discussion. Code frequency tables showing the number of occurrences each primary or subcode was applied to a transcript were generated. This allowed for the calculation of percentages of respondents that mentioned specific concepts, thus offering insight into broader trends in the data set.

## Results

Thirty-one individuals initially responded to advertisements for the study; 2 were ineligible as they responded “never,” when asked how often they looked at online recipes (6%) and 3 were ineligible because they did not qualify for free/reduced lunch (9%). Four individuals did not respond to contact from study staff after completing the screener (12%), and 2 individuals noted they were not interested upon learning more about the study (6%). Twenty participants (61% of those screened) completed the informed consent document, pre-interview survey and interview. No participants dropped out of the study once enrolled.

### Participant Characteristics and Cooking Behavior

Demographic, cooking, and recipe seeking characteristics of participants are shown in Table 1. Although all were invited to participate in this study regardless of sex, 100% of the final sample was female. Most were Hispanic White or Non-Hispanic Black (70%), and most had less than a college degree (65%). The majority of participants spent more than 30 minutes making dinner on average (85%). Online recipe seeking was common, with 40% of the sample interacting with online recipes most days of the week. While some mentioned using tablets or computers to find recipes online, 95% relied on their phones.

**Table 1.** Participant Characteristics.

	N (%)
Gender	
Female	20 (100)
Race/ethnicity	
Hispanic White	7 (35)
Non-Hispanic White	1 (5)
Hispanic Black	3 (15)
African American/Black	7 (35)
Other	2 (10)
Education level	
Less than high school	1 (5)
High school diploma	8 (40)
Some college	4 (20)
College grad or more	7 (35)
Time spent making dinner (average)	
<15 minutes	0 (0)
15-30 minutes	(15)
30 minutes-1 hour	(65)
>1 hour	4 (20)
Frequency of seeking online recipes	
4-7 days per week	8 (40)
2-3 days per week	10 (50)
≤1 day per week	2 (10)
Devices used to look for recipes	
Phone	19 (95)
Tablet	4 (20)
Computer/laptop	2 (10)

### Qualitative Findings

Cooking and online recipe seeking behaviors were qualitatively explored though analysis of the interview transcripts (n = 20). Three major themes emerged from the data: A) current cooking habits and environment, B) factors influencing healthy cooking, and C) role of the internet in home cooking. Table 2 includes selected primary codes and representative quotes to illustrate each of these themes. Quotes were selected to be representative of the concept and reflective of the broader data set. Quote selection was distributed across participants and aimed to include a diversity of ages and race/ethnicities of respondents, which are shown at the end of each quote for transparency in Table 2.

**Table 2.** Selected Codes and Quotes Encompassing Major Themes.

A) Current Cooking Habits and Environment		
*A1. Code: Usual meals*	*A2. Usual cooking methods.*	*A3. Kids meals*
Quote: Well, since my husband, he really enjoys Hispanic dishes--he’s from Mexico--so I really mostly cook a lot of Mexican dishes…like the traditional enchiladas, the red ones, taquitos, tacos, tostadas, a lot of guiso because they’re fast. And if it’s like cold weather, I’ll do like soups, like pozole or something._ Race/Ethnicity: Hispanic White (HW). Age: 31	I don’t do a lot of frying. I cook more so in my oven, and if I do anything fried, I use my air fryer a lot for stuff. I’m not using the oils_, African American (AA), 47	If I’m wanting to try a new recipe--my son is more picky than anyone else in the family, and my daughter doesn’t like to have a lot of dairy, so what I try to do is kind of stagger it so that the leftovers would be enough for them to have if my husband and I are going to try a new recipe, which we do probably one or two times a week at least. We try a new recipe. I make sure that there’s something my son can have instead on hand. And if there’s not, yes, I will go ahead and make something separate for them. _ Non-Hispanic White (NHW), 49
*A4. Cooking frequency and time*	*A5. Perceived confidence*	*A6. Meal planning*
I cook often. For the week, for the week, probably three times a week. When I cook, it lasts two days. I let it last two days, yeah, so probably three times a week I was cooking._ AA, 53	I don’t even have to look at the recipe, the written recipe. I can just look at the video and kind of get a feel for what all I need, how much I need just by looking at the video._ AA, 36	Since I’m a stay-at-home mom, I have to plan out these meals, and it helps when I wash the veggies earlier in the day and chop them up, and get it ready to prep_HW, 31

B) Factors That Influence Healthy Cooking		
*B1. Personal definition of a healthy diet*	*B2. Importance of healthy eating*	*B3. Barriers and facilitators to healthy cooking*
Healthy eating means to me a variation of getting a variation of nutrients and teaching your children how to do portion control and get a variety of things in their bodies_ AA, 47	Definitely [healthy eating is] important because I realize what it did to me as a child, like, oh my gosh--we didn’t have much money or much food, really, when I was growing up, so we would go and buy bags of chips and we’d buy cans of chili and we would just try to do the cheapest thing possible, like [brand] snacks and all that stuff. And I realized how that affected me going to school and affected how I ate as an adult. So I just try to keep it to where I’m telling him eating healthy is going to live a longer life._Hispanic Black (HB), 29	Lack of time. Like I forget to defrost something or I haven’t gone to the grocery store, or more importantly not defrosting something or not having enough time. If we get home late, for whatever reason, and then the kids are hungry, that makes it harder. So, sometimes, unfortunately, we’ll just pick something up, and that makes it harder, not having enough time_HW, 39

C. Role of the Internet in Home Cooking		
*C1. Goals for looking at online cooking content*	*C2. When/how access online recipes*	*C3. Preferred types of online recipe content*
Because I have to say, okay, this is what’s in my refrigerator; this is what is in my freezer and in my pantry. Sometimes it just draws a blank. I’m like this is all good stuff. Why can’t I think of anything. And I’m like, well, all right, I’ll just go on an adventure and look for a good recipe to go with what I have available._ HB, 39	Sometimes when I’m cooking in the kitchen, but then most time is when I’m relax, when I’m on my chill mode, afternoon time. Yeah, sometimes I hit the TV, or I look through my phone and check [for recipes]._ AA, 53	Yes. I usually [just] figure out what I want to cook. And then that day, I go to the store, and I’ll read like the recipe online, maybe Google. I like the little quick recipes like on TikTok or something like that. _AA, 31
*C4 factors that influence use of an online recipe*	*C5. Non-recipe online nutrition information seeking habits*	*C6. Learning to cook online*
For me, I like easy ones. I mean, kind of a variety also. During the summer, when we have more time, we might look up some international meals._Other race/ethnicity, 48	I have so many people that I follow, so I get a lot of nutritional tips from people, just different groups, you know, as far as even how to wash your fruits and vegetables properly. And I follow different people--and I didn’t even know anything about fruit pairing, different fruit pair well with other fruit. It’s a lot of information. Whatever I need, pretty much I can find on Pinterest._AA, 36	Pinterest for me has really helped me diversify my cooking and find recipes of dishes that I’ve tried at one point but never knew how to make and was able to make it now that I found it on Pinterest. It’s given me ideas. It’s given me shortcuts. It’s helped me figure out ingredients that you can replace for other stuff,_HW, 47

### Current Cooking Habits and Environment

The most common meals mothers reported cooking in this study were carbohydrate-based dishes (in which a grain-based product serves as the main component of the dish such as pasta or fried rice), (Table 2, Section A) including cultural foods from Mexico, the Caribbean, and Latin America (Table 2, A1). Some participants noted the importance of making meals adaptable for children by serving meal components separately or leaving off spices/ingredients children did not like and almost half (45%) noted cooking completely separate meals for their children at least some of the time due to picky eating (Table 2, A3). Many participants (55%) reported cooking meals using special equipment such as a slow cooker or air-fryer, which were hailed as beneficial for time management, health factors, and taste (Table 2, A2). Meal planning varied in the sample, with 20% specifically mentioning not planning meals, while 40% did a high level of meal planning by listing out dishes for the week (Table 2, A6). Most participants reported high cooking confidence (70%) and high cooking frequency (55%) when discussing their weekly home food preparation routines. Cooking frequency was sometimes tied to confidence as parents described building skills through practice, which in turn leads to a better ability to integrate new recipes found online (Table 2, A5).

#### Factors That Influence Healthy Cooking

**Figure 1.**
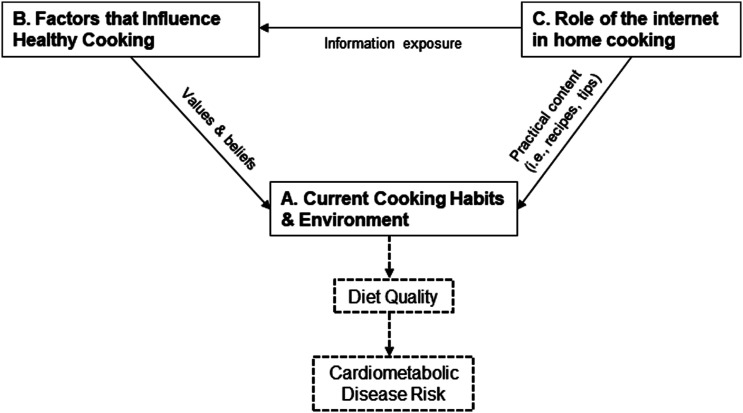
Network map of relationships between themes. Scheme illustrating the relationship between themes as identified in the data set. Dashed lines represent theoretical influence of cooking practices on diet quality and downstream nutrition-related disease risk.

There were several factors that emerged as relevant to shaping one’s ability to prepare healthy meals. (Table 2, Section 2B) Personal definitions of healthy eating varied in the sample but fell into four main, non-exclusive categories (Figure 1). The most common way participants discussed healthy eating was in terms of specific foods, such as avoiding processed foods (25%) or animal fats (45%), or eating fruits and vegetables (80%) Parents also discussed nutrients such as fat, carbohydrates and vitamins, but often with a focus on balance (Table 2, B1). Nearly 1/3^rd^ of participants (n = 6) described why healthy eating was important to them in the context of their role as caregivers (Table 2, B2). Several described being impacted by their lived experiences growing up with family members that had diet-related disease, or their current experiences coping with heart problems and weight issues. Time and competing responsibilities were mentioned as a barrier to healthy cooking by 14 participants (70%) (Table 2, B3). Family preferences were also a commonly mentioned factor that influenced healthy cooking. That is, in an environment where children and/or parents prefer vegetables or healthier meals, it is easier to prepare those foods. The opposite also emerged, if parents meet resistance to healthy dishes, they are less willing to prepare them. Over half of the sample (55%) described knowledge as a facilitator to healthy cooking, specifically in terms of having cooking skills (50%) and having access to recipes and nutrition information online (40%).

#### Role of the Internet in Home Cooking

Participants noted their goals when looking at online recipe content including: (Table 2, Section C) identifying recipes to cook at home (65%) (Table 2, C1), general inspiration (50%), and entertainment (25%). The most common scenarios in which parents were seeking online recipe information was when they were planning meals (60%) or in the kitchen preparing food (60%) (Table 2, C2). Casual viewing was common with half the sample indicating they browsed online recipes during downtime or weekends. Preferred resources for online recipes included search engines like Google (90%) and social media sites (75%). Most preferred recipe videos (75%), and written step-by-step recipes (60%), although some participants expressed annoyance at excessive narrative in written formats. Participants noted that they were more likely to try recipes if they came from a trusted site or influencer they were already familiar with and that had reliably fulfilled other criteria in the past (recipes that were easy, relatively fast, novel, liked by the family, used common ingredients) (Table 2, C4). Participants specifically described learning cooking and healthy eating skills through social media both by following health influencers (Table 2, C5) and by scrolling through content (Table 2, C6).

#### Relationships Between Major Themes

A network diagram was developed to explore the relationship between themes (Figure 1). This visualization brings together the emergent findings and reveals potential opportunities and barriers to leveraging online content for digital culinary medicine programming. The internet is a major resource for cooking and nutrition information among low-income mothers, and there are two paths of influence demonstrating the role of this new media on home cooking behaviors. First, respondents noted using practical, instructional information from online recipes and culinary tips to support skill development. Figure 1 conceptualizes this as the directional pathway from Theme C (role of the internet in home cooking) to Theme A (current cooking habits and environment). Second, respondents described how the internet exposes them to different types of people and information through social media by scrolling targeted pages (such as Pinterest boards) and following diet/nutrition influencers. This creates a learning environment wherein participants learn from multiple sources, re-shaping their beliefs and action related to healthy eating. This pathway is shown in Figure 1 as linking the Theme C (role of the internet in home cooking) to Theme B (factors that influence healthy cooking). Home cooking behavior changes as parents attempt to align their cooking habits with their values and beliefs about health-promoting behaviors and foods. This is shown on the figure as Theme B (factors that influence healthy cooking) linking to Theme A (current cooking habits and environment), which represents the actual practices that are occurring in participant’s kitchens, influencing diet quality and, in turn, downstream risk factors for diet-attributable disease.

## Discussion

This qualitative study, undertaken with parents from 6- to 11-year old children with low income, detailed several aspects of modern home cooking practices, perceptions of healthy eating and healthy cooking and the way in which home cooking is influenced by new media. Parents in our study expressed high usage and reliance on online resources for cooking information, and information exposure further influences cooking habits through impact on how people conceptualize healthy eating and healthy cooking. Through an examination of the relationships between cooking practices, new media, and health perceptions, we identified avenues for future culinary medicine efforts to support scale-up through digital programming.

### Implications of Findings for Culinary Medicine Research and Practice

These data inform potential opportunities for culinary medicine specifically, and health promotion messaging more broadly, and bring attention to barriers described by interview participants that may need to be considered when harnessing new media for public health (Table 3). High utilization of online recipes during both the cooking and meal planning process suggests a potential opportunity to leverage existing consumer interest in this media to promote recipes that are aligned with national nutritional guidelines. Putting existing recipes and instructional videos from established teaching kitchen programs online may support access to high quality online resources. This participation is critical, as existing online recipes are not necessarily designed to be healthy. Online recipe creators’ goals in making content vary, but recent analyses of existing recipe content suggest most are hedonistic in nature, designed to look and taste good, not necessarily maximize healthfulness.^35^ Additionally, online recipe content is largely unregulated, and our group has previously identified instances of health misinformation embedded into recipes on social media platforms.^36^ Acknowledging information quality as an issue is important when attempting to develop health promotion materials that rely on new media. One way to combat misinformation is by sharing accurate information first. Our findings emphasize the need for scientifically sound nutrition information to be communicated from reliable sources through new media channels.^37^

**Table 3.** Potential Culinary Medicine Opportunities in the Context of Online Food Media.

Pathway	Culinary Medicine Opportunity	Potential Barriers
C → A	Create practical tips and recipes based on dietary recommendations and informed by existing consumer preferences on food media	Competition with existing, more hedonistic content
C → A	Develop digital interventions (or extensions of teaching kitchen based programs) that leverage media for healthy cooking skill training	Adequate engagement in digital environment
C → B→ A	Disseminate scientifically accurate nutrition/diet information through social media/new media channels	Competition with sensationalist content/misinformation

The processes of identifying and using online recipes described by participants in this study suggest a reliance on content hubs (search engines, social media platforms) as opposed to specific websites. This approach exposes users to large amounts of information of varying quality. Interventions aiming to redirect users to specific health-focused sites and apps may have limited value, since parents are searching online for recipes and tips that fit their immediate needs and preferences, not necessarily searching for health-focused information.^38^ A better approach may be to teach parents specific ways to optimize the nutrition of the recipes they encounter online. Scalable culinary medicine programs that utilize new media to teach healthy cooking strategies have potential for this population, given their high utilization of online recipes and culinary content. However, digital interventions risk failure through low engagement.^39^ Many existing digital nutrition interventions integrate phone or in-person counseling, which may support motivation and engagement, but also adds cost to program design.^39,40^ Importantly, teaching kitchens and culinary medicine programs are increasingly popular in the United States. Thus, there may be potential to expand the reach of these programs by offering digital classes and/or resources as extensions to existing programmatic infrastructure. Both stand-alone digital culinary medicine programs, and those built to work with teaching kitchens will benefit from formative research with end users to understand the context in which people will use these types of technologies. This represents the first step of the user-centered design process which aims to support programs that are enjoyable and intuitive, thus supporting adoption and continued engagement.^41^ Findings from the current study, which detail the usage of online recipes in modern home cooking, will support future studies aiming to utilize new media in culinary medicine research and practice for parents with low income.

### Present Findings in the Context of Existing Literature

Our research represents some of the first in this area. Specific home cooking behaviors and the factors that influence their use have not been well studied despite increased investment in culinary medicine.^42–45^ It is critical to understand how new media influences home cooking behavior if we want to create scalable digital methods for culinary medicine. While few studies examining the use of online cooking content have been published, our results, wherein only 2 of the 31 individuals screened for our study indicated never looking at online recipes, are in line with findings from Tobey et al,^29^ who qualitatively examined recipe resources among mothers with low income as part of a larger study to refine a nutrition education smartphone application. The authors found that most (90%) of the 55 participants across 9 focus groups indicated using the internet for identifying recipes. Similar to our findings, Tobey et al also found parents often use online recipes to add variety to their cooking habits and get practical information for using ingredients they have on hand. While current research in this area is limited, it is important to consider that new media will likely continue to be a major resource for both practical cooking and nutrition information among families with low income as the digital divide between rich and poor continues to narrow,^46^ and internet and smartphone usage becomes even more ubiquitous in the population.^47^

### Strengths and Limitations

This study has several strengths including a predominantly minority sample of participants, with only one individual reporting race/ethnicity as non-Hispanic White. This is in line with our expectations as Hispanic and African American individuals are over-represented in poverty in Houston, Texas.^48^ Food security and SNAP/WIC participation was not collected in the sample; however, participants were only eligible for the study if they self-reported that their child was eligible for free or reduced school lunch. In Texas, the school lunch program utilizes the same income qualification as WIC (household income is ≤185% of the federal poverty line). We utilized teleconferencing methods to conduct interviews, which may have reduced barriers to participation for some individuals, while potentially limiting participation of those with less digital access. Only audio was used for the interviews, thus we may have missed some non-verbal or body language cues using this method. Importantly, although Hispanics are over-represented in poverty in urban Texas, interviews were only conducted in English which likely limited engagement with an important population (recent immigrants) that may be more likely to live in poverty. More research in Spanish and other languages is needed to better understand this phenomena among different subpopulations in the US.

## Conclusion

New media can play an important role in promoting healthier eating habits among families with low income through the creation of practical nutrition education content that is accessible, adaptable, and convenient. Future research on this topic may include cognitive task analysis through critical decision method^49,50^ interviews to gain detail and identify timelines, decision points, and factors influencing decision making related to online recipes. Information on low-income parents’ preferences for digital delivery of culinary medicine programs are also needed to inform the creation of enjoyable, intuitive tools that are aligned with user goals.

## Supplemental Material

Supplemental Material - Modern Home Cooking Practices, the Role of New Media, and Implications for Culinary Medicine: A Qualitative Study Among Mothers With Low Income Supplemental Material for Modern Home Cooking Practices, the Role of New Media, and Implications for Culinary Medicine: A Qualitative Study Among Mothers With Low Income by Margaret Raber, Maria Vazquez, Syeda Khan, Sahiti Myneni, and Debbe Thompson in American Journal of Lifestyle Medicine.

## Ethical Statement

### Ethical Approval

This study was reviewed and obtained ethics approval from the Baylor College of Medicine institutional review board (H50-324). Participants completed an informed consent document before taking part in study procedures.

## Data Availability

Data presented is primarily qualitative, interview transcripts will not be made available unless deidentified, by specific request, and with a formal data-sharing agreement in place to protect participant privacy. Subsequent use of data may require re-consenting of participants.

## References

[1] GBD 2017 Diet Collaborators . Health effects of dietary risks in 195 countries, 1990-2017: a systematic analysis for the global burden of disease study 2017. Lancet (London, England). 2019;393(10184):1958-1972. 30954305 10.1016/S0140-6736(19)30041-8 PMC6899507

[2] Vazquez CE Cubbin C . Socioeconomic status and childhood obesity: a review of literature from the past decade to inform intervention research. Curr Obes Rep. 2020;9(4):562-570. 32785878 10.1007/s13679-020-00400-2

[3] Eagle TF Sheetz A Gurm R , et al. Understanding childhood obesity in America: linkages between household income, community resources, and children’s behaviors. Am Heart J. 2012;163(5):836-843. 22607862 10.1016/j.ahj.2012.02.025

[4] Liu J Rehm CD Onopa J Mozaffarian D . Trends in diet quality among youth in the United States, 1999-2016. JAMA. 2020;323(12):1161-1174. 32207798 10.1001/jama.2020.0878 PMC7093765

[5] Rehm CD Peñalvo JL Afshin A Mozaffarian D . Dietary intake among US adults, 1999-2012. JAMA. 2016;315(23):2542-2553. 27327801 10.1001/jama.2016.7491 PMC6287627

[6] Banfield EC Liu Y Davis JS Chang S Frazier-Wood AC . Poor adherence to US dietary guidelines for children and adolescents in the national health and nutrition examination survey population. J Acad Nutr Diet. 2016;116(1):21-27. 26391469 10.1016/j.jand.2015.08.010 PMC4698034

[7] Hiza HA Casavale KO Guenther PM Davis CA . Diet quality of Americans differs by age, sex, race/ethnicity, income, and education level. J Acad Nutr Diet. 2013;113(2):297-306. 23168270 10.1016/j.jand.2012.08.011

[8] Clonan A Roberts KE Holdsworth M . Socioeconomic and demographic drivers of red and processed meat consumption: implications for health and environmental sustainability. Proc Nutr Soc. 2016;75(3):367-373. 27021468 10.1017/S0029665116000100 PMC4974628

[9] Daniels SR Pratt CA Hayman LL . Reduction of risk for cardiovascular disease in children and adolescents. Circulation. 2011;124(15):1673-1686. 21986774 10.1161/CIRCULATIONAHA.110.016170 PMC3751579

[10] Craigie AM Lake AA Kelly SA Adamson AJ Mathers JC . Tracking of obesity-related behaviours from childhood to adulthood: a systematic review. Maturitas. 2011;70(3):266-284. 21920682 10.1016/j.maturitas.2011.08.005

[11] Christoph MJ Larson NI Winkler MR Wall MM Neumark-Sztainer D . Longitudinal trajectories and prevalence of meeting dietary guidelines during the transition from adolescence to young adulthood. Am J Clin Nutr. 2019;109(3):656-664. 30831584 10.1093/ajcn/nqy333 PMC6408200

[12] Nepper MJ Chai W . Parents’ barriers and strategies to promote healthy eating among school-age children. Appetite. 2016;103:157-164. 27090341 10.1016/j.appet.2016.04.012

[13] Alkon AH Block D Moore K Gillis C DiNuccio N Chavez N . Foodways of the urban poor. Geoforum. 2013;48:126-135.

[14] Taillie LS . Who’s cooking? Trends in US home food preparation by gender, education, and race/ethnicity from 2003 to 2016. Nutr J. 2018;17(1):41. 29609591 10.1186/s12937-018-0347-9 PMC5881182

[15] Smith LP Ng SW Popkin BM . Trends in US home food preparation and consumption: analysis of national nutrition surveys and time use studies from 1965-1966 to 2007-2008. Nutr J. 2013;12:45. 23577692 10.1186/1475-2891-12-45 PMC3639863

[16] Farmer N Wallen GR Yang L Middleton KR Kazmi N Powell-Wiley TM . Household cooking frequency of dinner among non-hispanic black adults is associated with income and employment, perceived diet quality and varied objective diet quality, HEI (healthy eating index): NHANES analysis 2007-2010. Nutrients. 2019;11(9):2057. 31480746 10.3390/nu11092057 PMC6769568

[17] Mills S White M Brown H , et al. Health and social determinants and outcomes of home cooking: a systematic review of observational studies. Appetite. 2017;111:116-134. 28024883 10.1016/j.appet.2016.12.022

[18] Tiwari A Aggarwal A Tang W Drewnowski A . Cooking at home: a strategy to comply with U.S. dietary guidelines at no extra cost. Am J Prev Med. 2017;52(5):616-624. 28256283 10.1016/j.amepre.2017.01.017 PMC5401643

[19] Larson NI Perry CL Story M Neumark-Sztainer D . Food preparation by young adults is associated with better diet quality. J Am Diet Assoc. 2006;106(12):2001-2007. 17126631 10.1016/j.jada.2006.09.008

[20] Larson NI Story M Eisenberg ME Neumark-Sztainer D . Food preparation and purchasing roles among adolescents: associations with sociodemographic characteristics and diet quality. J Am Diet Assoc. 2006;106(2):211-218. 16442868 10.1016/j.jada.2005.10.029

[21] Berge JM MacLehose RF Larson N Laska M Neumark-Sztainer D . Family food preparation and its effects on adolescent dietary quality and eating patterns. J Adolesc Health. 2016;59(5):530-536. 27544460 10.1016/j.jadohealth.2016.06.007 PMC5606239

[22] van der Horst K Ferrage A Rytz A . Involving children in meal preparation. Effects on food intake. Appetite. 2014;79:18-24. 24709485 10.1016/j.appet.2014.03.030

[23] Ziauddeen N Page P Penney TL Nicholson S Kirk SF Almiron-Roig E . Eating at food outlets and leisure places and “on the go” is associated with less-healthy food choices than eating at home and in school in children: cross-sectional data from the UK national diet and nutrition survey rolling program (2008-2014). Am J Clin Nutr. 2018;107(6):992-1003. 29741556 10.1093/ajcn/nqy057 PMC5985724

[24] Cullen KW Bishop RG de Moor C . Fat practices and consumption among African-American adolescent boy scouts: the impact of meal source. Ethn Dis. 2002;12(2):193-198. 12019927

[25] Lachat C Nago E Verstraeten R Roberfroid D Van Camp J Kolsteren P . Eating out of home and its association with dietary intake: a systematic review of the evidence. Obes Rev. 2012;13(4):329-346. 22106948 10.1111/j.1467-789X.2011.00953.x

[26] Wolfson JA Leung CW Richardson CR . More frequent cooking at home is associated with higher healthy eating index-2015 score. Publ Health Nutr. 2020;23(13):2384-2394. 10.1017/S1368980019003549 PMC11374573 31918785

[27] Burton M Reid M Worsley A Mavondo F . Food skills confidence and household gatekeepers’ dietary practices. Appetite. 2017;108:183-190. 27693489 10.1016/j.appet.2016.09.033

[28] Garvin TM Chiappone A Boyd L , et al. Cooking Matters Mobile Application: a meal planning and preparation tool for low-income parents. Publ Health Nutr. 2019;22(12):2220-2227. 10.1017/S1368980019001101 PMC10260608 31084663

[29] Tobey LN Mouzong C Angulo JS Bowman S Manore MM . How low-income mothers select and adapt recipes and implications for promoting healthy recipes online. Nutrients. 2019;11(2):339. 30764537 10.3390/nu11020339 PMC6412388

[30] Wolfson JA Frattaroli S Bleich SN Smith KC Teret SP . Perspectives on learning to cook and public support for cooking education policies in the United States: a mixed methods study. Appetite. 2017;108:226-237. 27720707 10.1016/j.appet.2016.10.004

[31] Statistical Atlas . Overview of the Houston Area, Texas (Metro Area). Houston, TX: Statistical Atlas; 2018. https://statisticalatlas.com/metro-area/Texas/Houston/Overview Published 2018. Accessed April 19, 2021.

[32] Hennink MM Kaiser BN Marconi VC . Code saturation versus meaning saturation: how many interviews are enough? Qual Health Res. 2017;27(4):591-608. 27670770 10.1177/1049732316665344 PMC9359070

[33] Guest G Bunce A Johnson L . How many interviews are enough? An experiment with data saturation and variability. Field Methods. 2006;18(1):59-82.

[34] Fusch PI Ness LR . Are we there yet? Data saturation in qualitative research. Qual Rep. 2015;20(9):1408-1416.

[35] Cheng X Lin SY Wang K , et al. Healthfulness assessment of recipes shared on Pinterest: natural language processing and content analysis. J Med Internet Res. 2021;23(4):Article e25757. 33877052 10.2196/25757 PMC8097524

[36] Warner EL Basen-Engquist KM Badger TA Crane TE Raber-Ramsey M . The online cancer nutrition misinformation: a framework of behavior change based on exposure to cancer nutrition misinformation. Cancer. 2022;128(13):2540-2548. 35383913 10.1002/cncr.34218

[37] Ramachandran D Kite J Vassallo AJ , et al. Food trends and popular nutrition advice online - implications for public health. Online J Public Health Inform. 2018;10(2):Article e213. 30349631 10.5210/ojphi.v10i2.9306 PMC6194095

[38] Mohr DC Schueller SM Montague E Burns MN Rashidi P . The behavioral intervention technology model: an integrated conceptual and technological framework for eHealth and mHealth interventions. J Med Internet Res. 2014;16(6):Article e146. 24905070 10.2196/jmir.3077 PMC4071229

[39] Milne-Ives M Lam C De Cock C Van Velthoven MH Meinert E . Mobile apps for health behavior change in physical activity, diet, drug and alcohol use, and mental health: systematic review. JMIR Mhealth Uhealth. 2020;8(3):Article e17046. 32186518 10.2196/17046 PMC7113799

[40] Community Preventive Services Task Force . Nutrition and Physical Activity: Community-Based Digital Health and Telephone Interventions to Increase Healthy Eating and Physical Activity. Atlanta, GA: Centers for Disease Control; 2022. https://www.thecommunityguide.org/sites/default/files/assets/Nutrition-Physical-Activity-Digital-Health-Interventions-Community-508.pdf Published 2022. Accessed May 16, 2022.

[41] Wray TB Kahler CW Simpanen EM Operario D . User-centered, interaction design research approaches to inform the development of health risk behavior intervention technologies. Internet Interv. 2019;15:1-9. 30425932 10.1016/j.invent.2018.10.002 PMC6222087

[42] Fordyce-Voorham S . Identification of essential food skills for skill-based healthful eating programs in secondary schools. J Nutr Educ Behav. 2011;43(2):116-122. 21036670 10.1016/j.jneb.2009.12.002

[43] Polak R Phillips EM Nordgren J , et al. Health-related culinary education: a summary of representative emerging programs for health professionals and patients. Glob Adv Health Med. 2016;5(1):61-68. 26937315 10.7453/gahmj.2015.128 PMC4756781

[44] Raber M Chandra J Upadhyaya M , et al. An evidence-based conceptual framework of healthy cooking. Prev Med Rep. 2016;4:23-28. 27413657 10.1016/j.pmedr.2016.05.004 PMC4929050

[45] Raber M Wolfson J . The challenging task of measuring home cooking behavior. J Nutr Educ Behav. 2021;53(3):267-269. 33454197 10.1016/j.jneb.2020.11.012 PMC7954863

[46] Neuenschwander LM Abbott A Mobley AR . Comparison of a web-based vs in-person nutrition education program for low-income adults. J Acad Nutr Diet. 2013;113(1):120-126. 23092741 10.1016/j.jand.2012.07.034

[47] Auxier B Anderson M . Social Media Use in 2021. Washington, DC: Pew Research Center; 2021. https://www.pewresearch.org/internet/2021/04/07/social-media-use-in-2021/ Published 2021. Accessed February 15, 2022.

[48] U.S. Census Bureau . American Community Survey 1-Year Estimates. Houston, MD: U.S. Census Bureau; 2019. Retrieved from Census Reporter Profile page for Houston-The Woodlands-Sugar Land, TX Metro Area https://censusreporter.org/profiles/31000US26420-houston-the-woodlands-sugar-land-tx-metro-area/ Published 2019. Accessed September 29, 2021.

[49] Taylor HA . A critical decision interview approach to capturing tacit knowledge: principles and application. Int J Knowl Manag. 2005;1(3):25-39.

[50] Barber T Sharif B Teare S , et al. Qualitative study to elicit patients’ and primary care physicians’ perspectives on the use of a self-management mobile health application for knee osteoarthritis. BMJ Open. 2019;9(1):Article e024016. 10.1136/bmjopen-2018-024016 PMC6361338 30782723

