# Supplementary material for: Modern Home Cooking Practices, the Role of New Media, and Implications for Culinary Medicine: A Qualitative Study Among Mothers With Low Income
Source: Am J Lifestyle Med. 2023 Aug 24;20(2):233–43. doi: 10.1177/15598276231197181 (PMC12766009; doi:10.1177/15598276231197181)
Supplement: Supplemental Material - Modern Home Cooking Practices, the Role of New Media, and Implications for Culinary Medicine: A Qualitative Study Among Mothers With Low Income [file sj-pdf-1-ajl-10.1177_15598276231197181.pdf]

Supplementary Material 1: Cooking Survey Text

Survey Description

We are using this survey to collect opinions about online cooking information in order to create new healthy eating programs for families with children. This survey will be followed by a phone or online (e.g. Zoom) interview, which will ask you for a bit more information about your answers.

Let's get started! First, we are going to ask about where you find online recipes, and how you use them.

Where do you find recipes online? (check all that apply)

- Search Engine (Google)
- Social Media (Facebook, Twitter, Instagram, TikTok, Pinterest etc.)
- Specific Websites / Blogs
- Email Newsletters / Listserves
- Friends
- Other (please explain) _________

How often do you see recipes online?

- Every day
- Most days
- Sometimes (2/3 days a week)
- Rarely (once a week or less)

What types of online recipes/cooking information do you prefer (check all that apply)?

- Short videos (such as those on TikTok, Instagram Reels, Tasty)
- Longer videos (such as those on Food Network)
- Photos alone (such as those on Instagram, Facebook)
- Blogs
- Emails
- Step by step photo recipes
- Step by step written recipes
- Other (please explain)_______________

When you cook at home, how frequently do you use recipes that you found online?

- 1 – Never use
  2 – Almost never use
  3 – Occasionally/sometimes use
- 4 – Use almost every time
  5 – Use every time / Frequently use

Which device do you normally use to look at online recipes?

- Cell phone
- Tablet
- Computer/laptop
- Other (please explain)________

Please share some of your favorite online cooking videos/recipes/websites with us by typing your favorite sites in the box below, or cutting and pasting links to your favorite recipes . (You can add as many as you like!):____________________

How much time do you normally spend making dinner on a weeknight?

- Less than 15 minutes
- About 15 – 30 minutes
- About 30 minutes to an hour
- More than an hour

Indicate how much you agree with the following statements:

(disagree / neither / agree)

a. I enjoy trying new recipes
b. I prefer recipes that are fast
c. I prefer recipes that are healthy
d. I plan meals in advance
e. When I prepare a meal, I make enough so I will have leftovers
f. Cost of ingredients is important when I choose what to make for dinner.
g. I make separate meals / dishes for my kids
h. I can adapt a recipe if I am missing an ingredient
i. I look at recipes or videos online while I am cooking
j. I prefer to print recipes out

When you cook your evening meal at home, is there a food you almost always try to include (check all that apply)?

- Bread or Tortillas
- Rice or Grains
- Vegetables
- Meat or Fish

Supplementary Material 1: Cooking Survey Text

The next few questions will ask about you.

What is your current age?

What is your gender?

- Male
- Female
- Other Non-Binary

What category best describes your ethnic background?

- Hispanic/Latino
- Not Hispanic/Latino

What category best describes your race? Choose all that apply:

- American Indian/Alaskan Native
- Asian
- Black/African American
- Native Hawaiian/Pacific Islander
- White
- Other
-

What is your highest level of education?

- Less than High School Never received high school diploma/GED
- High School Diploma/GED/ Technical School
- Some College
- College Degree
- Advanced Degree

How many children under 18 live in your household? ____

What are the ages of your children under 18 (please separate with commas)? ________
